# Effects of secondhand smoke on the birth weight of term infants and the demographic profile of Saudi exposed women

**DOI:** 10.1186/1471-2458-13-341

**Published:** 2013-04-15

**Authors:** Hayfaa A Wahabi, Rasmieh A Alzeidan, Amel A Fayed, Ahmed Mandil, Ghadeer Al-Shaikh, Samia A Esmaeil

**Affiliations:** 1 Sheikh Bahamdan Research Chair of Evidence-based Healthcare and Knowledge translation, College of Medicine, King Saud University, P.O Box 102799, Riyadh, 11685, Kingdom of Saudi Arabia; 2 Department of Family and Community Medicine, College of Medicine, King Saud University, Riyadh, Kingdom of Saudi Arabia; 3 King Saud Ben Abdul Aziz University for Health Sciences, Riyadh, Kingdom of Saudi Arabia; 4 High Institute of Public Health Alexandria University, Alexandria, Egypt; 5 Obstetrics and Gynecology Department, College of Medicine, King Khalid University Hospital, Riyadh, Kingdom of Saudi Arabia

**Keywords:** Second hand smoke, Newborn anthropometry, Saudi Arabia

## Abstract

**Background:**

Maternal exposure to tobacco smoke during pregnancy is associated with detrimental effects on the mother and the fetus including; impaired fetal growth, low birth weight and preterm delivery. In utero exposure to tobacco is implicated in the etiology of many adults’ diseases including obesity, diabetes and hypertension.

The objectives of this study were to evaluate the effects of Secondhand Tobacco Smoke (SHS) exposure on newborns’ anthropometric measurements and to compare the demographic profile of the women exposed to SHS to those who were not.

**Method:**

This is a retrospective cohort study investigating the effects of SHS during pregnancy on newborns’ anthropometry. Women who self-reported SHS exposure were compared with those not exposed. The primary outcomes were birth weight, newborn length and head circumference. Univariate analysis and multivariate regression analysis were performed. Adjusted differences with 95% confidence intervals were calculated.

**Results:**

Mothers exposed to SHS constituted 31% of the cohort. The mean birth weight of infants of exposed mothers was significantly lower by 35 g, 95% CI: 2–68 g, (*P* = 0.037) and the mean length was shorter by 0.261 cm, 95% CI 0.058-0.464 cm, (*P* = 0.012) compared to the infants of unexposed mothers. Women exposed to SHS, were younger, of lower parity and more likely to be illiterate than those who were not exposed in addition, exposed women were less likely to be primiparous.

**Conclusion:**

The prevalence of exposure of Saudi pregnant women to SHS is high at 31% and it is associated with reduced birth weight, and shorter length of the newborn.

## Background

Maternal smoking during pregnancy is associated with detrimental effects on the mother and the fetus including; impaired fetal growth, low birth weight, preterm delivery and increased neonatal and infant mortality rate [1-4]. Similar effects were reported for pregnancies exposed to secondhand smoke (SHS) [5]. Moreover in-utero exposure to tobacco is rooted in the etiology of many adults’ diseases including obesity, diabetes and hypertension [6].

Similar to other reproductive health problems, there is paucity of information about the magnitude of tobacco smoking (both active and SHS) during pregnancy in Arab countries including the Kingdom of Saudi Arabia (KSA). The few published reports about tobacco smoking and SHS exposure during pregnancy are limited by either the small number of participants [7] or the difference in culture and social norms between geographical areas in the same country or between one country and another which limit generalization of results [8]. Moreover some of these studies are outdated considering the major socioeconomic and life style changes in some counties such as the KSA, during last few decades [9].

The latest country survey in KSA showed that the prevalence of tobacco use among males is estimated at 24% and at 1% among females [10]. Data about tobacco use in pregnancy was reported in one study [9]. The study confirmed the adverse effect of SHS exposure on birth weight; however it was not designed to estimate the prevalence of SHS exposure in pregnancy or the maternal knowledge or attitude towards SHS [9].

KSA is one of the wealthiest countries in the Middle East; the Gross National per Capita Income is $22,750 in 2009 [11]. Due to increased expenditure on healthcare there is rapid development in health services provision associated with improved socio-economic conditions. The Saudi Ministry of Health has implemented a national tobacco control program which is based on the WHO framework Convention for Tobacco Control and WHO-recommendation of the MPOWER strategies [12].

The implementation of any program aiming to reduce in-utero exposure to tobacco is dependent on the demographic profile of the pregnant women in the population and the nature of exposure to smoke, as being active smokers themselves or exposed to SHS.

This study was designed to:

1. Investigate the prevalence of tobacco use and SHS exposure among pregnant women in King Khalid University Hospital (KKUH)

2. Evaluate the effects of SHS exposure on the newborn biometric measurements (birth weight, length and head circumference)

3. Compare the demographic profile of the women exposed to SHS to those who were not exposed.

## Methods

This is a retrospective cohort study, conducted at postnatal ward of KKUH in KSA. KKUH is a tertiary referral center; which includes a neonatal intensive care unit (NICU) and in vitro fertilization unit. The obstetrics department provides services for 3500–4000 deliveries per year.

The study was designed to investigate the effects of maternal exposure during pregnancy to SHS on the newborns’ anthropometric measurements (birth weight, length, head circumference) as primary outcomes, in addition we investigated the association of SHS with the following secondary outcomes; APGAR scores at 1 and 5 minutes, low birth weight (LBW) < 2.5 kg and admission to NICU.

After reviewing the literature [13], the sample size was based on an expected difference of 30 g of birth weight between infants of women exposed and unexposed to SHS; the birth weight of Saudi newborn was reported to be around 3.100-3.200 kg [14]. At 95% significance level (α =5%) and a power of 80% (β =20%), the minimal sample size required to reject the null hypothesis was 2782 for both groups. To account for missing data the study was conducted over 12 months between the 1^st^ of July 2011 and 30^th^ of June 2012. Consecutive women who consented to join the study and met the inclusion criteria were enrolled. The inclusion criteria were:

1. Women with singleton pregnancy.

2. Term delivery (≥ 37 gestation week counted from the last menstrual period and/or early ultra-sound scan).

3. Women who did not smoke during the index pregnancy and were exposed to SHS (study group).

4. Women who did not smoke during the index pregnancy and were not exposed to SHS (control group).

We excluded from this study women with unknown smoking status.

Data were collected using a predesigned data collection sheet from women in the postnatal ward following delivery and before discharge from the hospital, by nurses who were trained to collect the data. Women who met the inclusion criteria and consented to the study were asked about their exposure to SHS which was defined as occurring when a woman, who did not smoke at all whilst pregnant, lived with a household member (husband, son, daughter or other relatives) who reported smoking during the index pregnancy. We did not assess occupational exposures. In addition participants were asked about their level of education (illiterate, schooling, university or above) and if they work for pay.

Data collected from the delivery records included gestational age at delivery, APGAR scores at 1 and 5 minutes, weight, length and head circumference of newborn and admission to NICU. Researchers who collected the data from the records were blinded to the smoking status of the mother. We compared the birth weight, length and head circumference, of infants of mothers who were exposed to SHS to those of mothers who were not exposed. In addition we compared the frequency of occurrence of LBW (< 2.5 kg) and APGAR scores at 1 and 5 minutes between the two groups.

The data collected from the antenatal records included; maternal age, gravidity, parity, maternal height and weight recorded during the first antenatal visit, from which, body mass index (BMI) was calculated according to the following equation; BMI = weight (kg)/height (m)^2^[15], in addition to antenatal events including the occurrence of preeclampsia defined as blood pressure ≥140/90 mm Hg after 20 weeks gestation and ≥ 0.3 g proteinuria/day, pregnancy induced hypertension defined as blood pressure ≥140/90 mm Hg after 20 weeks gestation without proteinuria and gestational diabetes (GDM) as per antenatal record diagnosis. These variables were extracted and analyzed as confounders due to their known influence on newborn anthropometry.

The ethical approval for the study was granted by the college of medicine, King Saud University Institutional Review Board, before the commencement of the study.

### Statistical analysis

Statistical analyses were performed using SPSS, version 18.0 (SPSS Inc., Chicago, IL, USA). Descriptive statistics were computed for non-smoking pregnant women exposed and unexposed to SHS. Univariate analyses were performed to compare the birth weight, infant’s length and head circumference between the two groups as well as to evaluate the baseline characteristics between the groups which we considered as confounding factors. Chi-squared was used to compare dichotomous outcomes and Student’s t- test was used to compare continuous outcomes. Stepwise logistic regression models were used to adjust for potential confounders including maternal age, parity, BMI, GDM and gestational age (37–42 weeks). *P* value of < 0.05 was considered statistically significant.

## Results

During the study period there were 3766 deliveries of them 3 women self-reported active smoking and 3426 met the inclusion criteria and consented to the study. 1085 (31.7%) women self-reported exposure to SHS while 2341 (68.3%) did not report such exposure. The demographic characteristics of the women exposed and not exposed to SHS are shown in Table 1. Of the study population 3241 (94.6%) were Saudi. Women exposed to SHS, were younger, of lower parity and more likely to be illiterate than those who were not exposed. However exposed women were less likely to be primiparous.

The results of the pregnancy outcomes of the exposed and non-exposed women are shown in Table 2. Infants of women who were exposed to SHS had significantly less birth weight, and were significantly shorter than infants of non-exposed women. The mean head circumference of the infants of exposed mothers was smaller than that of infants of unexposed mother; however the difference did not reach statistical significance. Similarly, the frequency of low birth weight infants (<2.5 kg) was higher among infants of exposed mothers but the difference did not reach statistical significance.

Table 3 summarizes the results of the stepwise multivariate regression analysis for birth weight. After adjustment for confounding factors, the mean birth weight of infants of mothers exposed to SHS remained significantly lower by 35 g, 95% confidence interval (CI) 2-68 g, (*p* = 0.037), compared to the birth weight of infants of unexposed mothers (Table 3). Similarly, the mean length of infants of mothers exposed to SHS was significantly shorter compared to those of mothers who were not exposed, by 0.261cm, 95% CI, 0.058-0.464 cm, (*p* = 0.012) (Table 4).

## Discussion

We found that SHS constitutes a public health problem as more than 31% of women included in this study reported exposure to domestic SHS, with documented adverse effects on the birth weight and the newborn length.

Similar to maternal smoking during pregnancy, many studies confirmed that, exposure to SHS has adverse effects on the mother and the fetus [16,17]. The suggested mechanisms for the reduced birth weight are the negative effects of nicotine and cotinine on the placental development and on its function of oxygen transfer to the fetus [18,19]. Like other studies we did not find significant difference in the rate of LBW between infants of mothers exposed to SHS and those who were not [16,17]. Nevertheless this finding does not disprove the adverse effects of smoking on the infants of normal birth weight as demonstrated by an earlier study, which showed that the mortality curve of infant exposed to smoking at any measure of birth weight is higher than for unexposed ones [20]. We believe that the results of the effects of SHS exposure on the newborn anthropometric measurements, in this study, are reliable because we adjusted for multiple confounding factors which are known to influence the birth weight (Table 3). Unlike previous reports [21,22] we consider GDM a confounding factor due to the reported high prevalence of the condition among Saudi pregnant women [14] which was further confirmed by the high prevalence of 15%, found in this study (Table 1).

The demographic profile of the pregnant women exposed to SHS in this study provides valuable information for future interventions to reduce SHS exposure in the household. It confirmed that most of the respondents to the survey were Saudis; hence any intervention based on the information from this study would be primarily directed towards the stable population of the country. Of paramount importance is the high level of literacy among the participants, which would facilitate the use of written and electronic information to increase the awareness and the knowledge about the hazards of SHS exposure in the household. However the high literacy level among the respondents of this study may not reflect the situation in rural areas of the country as differences in literacy levels were reported between urban and rural areas by other investigators [23].

Less than 20% of the respondents were working for pay, which makes SHS exposure in the workplace unlikely for most pregnant women, compared to household exposure, considering that there is gender segregation in most of the workplaces and that only 1% of the Saudi women were reported to smoke [10].

**Table 1:** **Demographic characteristics of non**-**smoking women by exposure to SHS**

**Characteristic**	**Exposure to SHS**				***P *value**
		**Total number 1085 Yes**		**Total number 2341 No**	
Maternal age (years)		28.83 ± 6.11		29.60 ± 6.20	**0**.**001**
Primiparous		674 (66.8)		1541(71.7)	**0**.**005**
Parity		2.78 ± 2.08		3.02 ± 2.20	**0**.**003**
Gravidity		3.31 ± 2.62		3.53 ± 2.67	**0**.**03**
BMI (Kg/m^2^)		29.69 ± 6.14		29.48 ± 5.98	0.37
Gestational diabetes mellitus	1007*	136 (13.5)	2150*	329 (15.3)	0.18
Pregnancy induced hypertension	1000*	11(1.1)	2153*	28 (1.3)	0.62
Preeclampsia	1000*	4 (0.4)	2285*	16 (0.7)	0.25
Level of education					
illiterate	1000*	32 (3.2)	2230*	29 (1.3)	<**0**.**001**
schools	1007*	548 (54.4)	2151*	983 (45.7)	
University and above	1007*	428 (42.5)	2151*	1138(52.9)	
Work status					
housewife	1009*	771 (76.4)	2149*	1610(74.9)	0.24
student	1009*	104 (10.3)	2145*	266 (12.4)	
employee	1007*	134 (13.3)	2157*	274 (12.7)	

*Total number in the variable excluding missing data.

Data are n (%) or means ± standard deviation.

Statistical tests used were student *t* test, chi-square test, and Fisher exact test.

SHS = Secondhand smoke. BMI = Body mass index.

In this study only three women reported active smoking during pregnancy while more than 31% reported SHS and more than 80% of the participants reported their husbands as the main source of SHS in the household. These results are consistent with the findings from 31 developing countries [24]. The implications of these results are that any intervention for reducing SHS exposure in the household should be directed to both parents, rather than to the mother only, and that interventions implemented for smoking cessation may indirectly reduce the exposure to SHS by decreasing the number of smoking males.

Studies from countries in Asia, which evaluated maternal knowledge about SHS health hazards to the mother and the baby, showed that most respondents were aware of the general harmful effects of SHS, however very small number were aware of the specific effects of SHS on the fetus [23]. Knowledge of the pregnant women of the health hazards of SHS to their pregnancies is imperative for reducing SHS exposure. Barbour et al. reported that women’s knowledge about the harmful effects of SHS to the baby was stronger motivator for smoking cessation, than knowledge about the effects of SHS on the mother who smokes [25]. Similar findings were reported by others, on parents’ attitude towards indoor smoking when they were aware of the harmful effects of SHS on the children’s health [26,27]. We believe such motivation should be utilized to encourage parents to adopt the practice of avoiding SHS in the household by increasing their knowledge about the harmful effects to all the children in the household including the unborn child.

**Table 2:** **The results of the perinatal outcomes in non**-**smoking women by exposure to SHS**

**Outcome**	**Exposure to SHS**				***P *value**
		**Total number (1085) Yes**		**Total number (2341) No**	
Birth weight		3.15 ± 0.46		3.21 ± 0.46	**0**.**002**
Baby’s length		49.62 ± 3.09		49.87 ± 2.48	**0**.**014**
Head circumference		34.05 ± 1.59		34.14 ± 1.73	0.17
Apgar at 5 min		8.92 ± 0.64		8.91 ± 0.66	0.75
Apgar at 1 min		7.73 ± 0.86		7.75 ± 0.82	0.66
Low birth weight (<2500 gms)	1000*	54 (5.4)	2152*	99 (4.6)	0.36
NICU	1000*	45 (4.5)	2155*	97 (4.5)	0.79

*Total number in the variable excluding missing data.

Data are n (%) or means ± standard deviation.

Statistical tests used were student *t* test, chi-square test, and Fisher exact test.

SHS = Secondhand smoke. NICU = Neonatal intensive care unit.

**Table 3:** **Regression model for birth weight at term** (**37 weeks or more**)

	**Adjusted difference**	**95% CI**	**p-value**
Gestational age	0.099	0.086 to 0.113	<0.0001
BMI	0.010	0.007 to 0.012	<0.0001
Maternal age	0.005	0.002 to 0.009	0.005
Level of education	0.058	0.028 to 0.88	<0.001
Gestational diabetes	0.066	0.022 to 0.11	0.002
Parity	0.012	0.001to 0.022	0.032
SHS	-0.035	-0.068 to -0.002	0.037

Statistical tests used were student *t* test, chi-square test, and Fisher exact test.

SHS = Secondhand smoke. BMI = Body mass index.

Although public health interventions such as mass media campaigns, legislative banning of smoking in public places and increasing tobacco prices and taxes were proven to be effective in reducing the prevalence of tobacco smoking and SHS exposure in public places, such interventions were not only ineffective in reducing exposure to SHS in the household [28] but might even increase the risk of exposure to SHS at home [29]. Similarly the use of an air cleaner was proven to be effective in reducing the concentration of particulate matter with improvement in clinical symptoms of asthmatic children; however this intervention did not reduce exposure to SHS [30].

Of the clinical interventions, physician counseling was reported to be an effective intervention in reducing exposure to SHS among pregnant women; however the effectiveness of this intervention was not linked to improvement of pregnancy outcomes [31]. On the other hand results from systematic reviews did not support the effectiveness of brief counseling in reducing household exposure to SHS [32].

**Table 4:** **Regression model for baby**’**s length at term** (**37 weeks or more**)

	**Adjusted difference**	**95% CI**	**p-value**
Gestational age	0.352	0.273, 0.432	<0.0001
BMI	0.028	0.011, 0.044	0.001
Maternal age	0.021	0.005, 0.037	0.011
SHS	-0.261	-0.464, -0.058	0.012

Statistical tests used were student *t* test, chi-square test, and Fisher exact test.

BMI = Body mass index. SHS = Secondhand smoke.

Recently, the use of personalized data of the house air quality has been investigated and proven effective in motivating smoking mothers, with small children, to change their smoking behavior with positive impact on the air quality of the house [33,34].

Our study has confirmed the high prevalence of exposure of pregnant women to SHS and it documented a biological harmful effect of SHS on the newborn in a Saudi community; in addition it has outlined the demographic profile of the pregnant Saudi women who were exposed to SHS. The results of this study would help in planning an effective program for reducing exposure to SHS for pregnant women in KSA.

We are aware of the limitations of this study including that the exposure to SHS was based on women’s self-report without the use of biomarker to verify exposure. Another limitation is that we did not quantify the exposure to SHS by the number of hours the mother exposed; or the number of smokers in the in the family, both factors were proven to increase the health risks of SHS [24] hence we did not report a dose response relationship between exposure to SHS and pregnancy outcomes; however due to the self-reported design of the study and the possibility of recall bias, a dose response might not have been verified.

## Conclusion

The prevalence of exposure of Saudi pregnant women to SHS is high at 31% and it is associated with reduced birth weight, and shorter length of the newborn.

## Competing interest

The authors declare that they have no competing interest.

## Authors’ contribution

HW conceived the idea of the study, was responsible for writing the final study manuscript. AF was responsible for the statistical analysis; she participated in writing the draft of the manuscript. RZ, AM, GA participated in writing the draft of manuscript. All authors reviewed and approved the final manuscript.

## Pre-publication history

The pre-publication history for this paper can be accessed here:

http://www.biomedcentral.com/1471-2458/13/341/prepub
